# Telomere Dynamics Throughout Spermatogenesis

**DOI:** 10.3390/genes10070525

**Published:** 2019-07-12

**Authors:** Heather E. Fice, Bernard Robaire

**Affiliations:** 1 Department of Pharmacology and Therapeutics, McGill University, Montreal, QC H3G 1Y6, Canada; 2 Departments of Obstetrics and Gynecology, McGill University, Montreal, QC H4A 3J1, Canada

**Keywords:** male germ cells, spermatogenesis, sperm, telomeres, chromatin, reproductive aging

## Abstract

Telomeres are repeat regions of DNA that cap either end of each chromosome, thereby providing stability and protection from the degradation of gene-rich regions. Each cell replication causes the loss of telomeric repeats due to incomplete DNA replication, though it is well-established that progressive telomere shortening is evaded in male germ cells by the maintenance of active telomerase. However, germ cell telomeres are still susceptible to disruption or insult by oxidative stress, toxicant exposure, and aging. Our aim was to examine the relative telomere length (rTL) in an outbred Sprague Dawley (SD) and an inbred Brown Norway (BN) rat model for paternal aging. No significant differences were found when comparing pachytene spermatocytes (PS), round spermatids (RS), and sperm obtained from the caput and cauda of the epididymis of young and aged SD rats; this is likely due to the high variance observed among individuals. A significant age-dependent decrease in rTL was observed from 115.6 (±6.5) to 93.3 (±6.3) in caput sperm and from 142.4 (±14.6) to 105.3 (±2.5) in cauda sperm from BN rats. Additionally, an increase in rTL during epididymal maturation was observed in both strains, most strikingly from 115.6 (±6.5) to 142 (±14.6) in young BN rats. These results confirm the decrease in rTL in rodents, but only when an inbred strain is used, and represent the first demonstration that rTL changes as sperm transit through the epididymis.

## 1. Introduction

The male germline is biologically unique in many ways, ranging from cellular structures to chromatin packaging and enzymatic activity. Telomeres are no exception to this statement, with telomere dynamics in male germ cells being distinctly different from those of somatic cells. Telomeres are 5′-TTAGGG-3′ repeat sequences that cap the ends of chromosomes to give the genome protection and stability from progressive shortening after DNA replication caused by the incomplete replication of the 5′ end by DNA polymerase [1]. The progressive shortening of telomeres due to the end-replication problem can be mitigated by the enzyme telomerase; it maintains telomeres by the addition of new repeats. Containing both a protein (TERT) and an RNA template (TERC), the enzyme functions as a reverse transcriptase synthesizing a single strand of telomeric DNA complementary to TERC onto the 3′ overhang. This newly synthesized telomeric DNA strand is then used for lagging strand synthesis by DNA replication machinery [1]. Alternative lengthening of telomeres (ALT) by homologous recombination is another mechanism by which telomeres and subtelomeric regions are able to retain their length in the absence of active telomerase [2].

The existing literature presents data on the length and spatial arrangement of telomeres, and the activity of telomerase within germ cell nuclei as spermatogenesis progresses [3]. Beginning in spermatogonia, telomerase is most active and telomere length is hypothesized to be shorter relative to fully mature spermatozoa [4]. The telomeres at this stage are randomly positioned; however, during mitosis, they align to either pole of the cell in preparation for cytokinesis. In spermatocytes, telomerase levels are high and telomeres follow a similar alignment once meiotic events are initiated. Round spermatids have similarly high levels of telomerase at the onset of spermiogenesis; however, these levels decrease as the cells become transcriptionally inactive during chromatin compaction [4]. At this stage, fluorescence in situ hybridization (FISH) experiments have also shown that telomeres spread randomly throughout the cell nucleus. Interestingly though, FISH experiments often display a reduced number of telomeres due to their apparent dimerization. This has been shown as the number of telomeres present at the final stages of spermiogenesis is half of the expected number, suggesting that they are co-localizing [5,6,7]. Some hypotheses have been put forth about the nature of this interaction, and through FISH experimental staining for p and q arms of chromosomes 3 and 6, it appears that the telomeres of each chromosome bind to each other in a loop-like fashion [7]. Throughout spermiogenesis and epididymal transit, germ cells undergo dramatic chromatin repackaging. There is a gradual replacement of most histone-bound nucleosomes first with transition proteins and then with protamines, to form a tight toroidal conformation [8]. This repackaging event does not completely void the cell of histones, and approximately 10–15% of histones are retained in human sperm [9,10,11], while in rodents, only 1–2% of histones are retained [11,12]. Fully mature sperm maintain dimeric telomeres, as shown in round spermatids, though they contrast with earlier germ cells as little to no telomerase activity has been observed [13]. Telomere length in spermatozoa is longer than in somatic cells and has been measured at approximately 6–20 kb in humans [14,15,16,17,18]. Spermatozoa also appear to have a specific organization of telomeres, with the telomeric regions of chromatin found toward the nuclear periphery or bound to the nuclear membrane. This observation has been shown for many species, including humans, rodents, primates, and bovine [14,19]. It has been postulated that the combination of histone-bound telomeres and their arrangement at the nuclear periphery serves a functional role after fertilization as these sites are more readily accessible by the oocyte for pronuclear formation [6,20].

A central current issue in male germ cell telomere biology is whether telomere length can be used as a biomarker for sperm quality and fertility. The parameters set by the World Health Organization (WHO) used to assess male fertility do not capture information about sperm chromatin quality [21]. Although measuring sperm DNA integrity is considered an important endpoint [22], many of the methods have been classically challenging in a clinical setting as they require a high level of technical expertise. As a result, there is a demand for a quick reproducible test that would examine a new sperm parameter. Telomere length is a desirable measure, as preliminary studies are beginning to suggest links between fertility outcomes and sperm telomere length. However, there is some controversy in this field regarding which methods measure telomere length in a reliable and accurate way. The methods employed include Southern blotting, fluorescence in situ hybridization, and the quantitative polymerase chain reaction (qPCR). Both Verhulst [23] and Eisenberg [24] have discussed the issues as they relate to each method’s reliability, pointing out the inherent cost–benefit analysis that must be done when deciding on a method. When assessing telomere length as a biomarker for fertility in humans, it would be most appropriate to use qPCR as it is relatively simple, inexpensive, and allows for a high-throughput analysis of many samples.

As previously mentioned, preliminary data on the links between sperm telomere length and well-established fertility parameters are beginning to emerge [25]. Several studies have found an association between a shorter telomere length and infertility or oligozoospermia [26,27,28,29,30], but not with classical WHO semen parameters. Interestingly, Garolla et al. found a positive association between sperm telomere length and protamination status [31]. This finding suggests that an error in chromatin packaging results in telomere dysregulation in mature sperm. Additionally, more loosely packaged chromatin could result in an increase in exposure to reactive oxygen species.

There are many factors known to increase male factor infertility, including smoking, alcohol, toxicant exposure, and being overweight [32]. These lifestyle factors, in addition to the aging process, greatly increase the presence of reactive oxygen species; several studies have found an association between these lifestyle factors and disrupted sperm telomere integrity [33,34]. Telomeres are particularly susceptible to oxidative damage as they are highly rich in guanine, allowing for the oxidization to 8-oxo-2’-deoxyguanosine (8-oxo-dG) [35]. In vitro results suggest that oxidative insult results not only in disrupted telomere integrity, but also in telomere shortening [36]. Additionally, the retention of histones in telomeric regions makes these regions more sensitive to oxidative insult [20]. The DNA damage that may be incurred from these oxidative insults can further lead to telomeric instability and telomere–telomere interactions may be lost [37].

Telomere length decreases in somatic cells with advanced age, but there are varying species-dependent effects on sperm telomere length. In studies examining telomere length in mice, the trend with advanced paternal age is a decrease in telomere length, similar to that seen in somatic cells [38]. However, when similar studies were done using human sperm, the telomere length appeared to increase with age [16,39]. There are two main hypotheses addressing the potential cause of telomere lengthening in species with longer life spans. The first is that because telomerase is active in spermatogonia and throughout spermatogenesis, it has ample time to act and build on telomeres as the pool of stem cells is aging. The second is that there is a selection of germ cells for those with the longest telomeres over the course of a man’s lifespan, resulting in only those with long telomeres remaining at an advanced age [40].

Telomere homeostasis may exist, where there is a balance for the optimal telomere length. When the telomeres are dysregulated, meiosis can be more error-prone, with chromosome segregation being incomplete and higher rates of aneuploidy [41]. Supporting this hypothesis, Cariati et al. have shown data that there are pregnancy failures when male partners have short telomeres [28]. It is also interesting to note that these studies have explored the association between sperm telomere length and offspring leukocyte telomere length. Few studies have directly studied both sperm telomere length and offspring telomere length; however, in rodents, birds, primates, and humans, there is a clear paternal age effect on telomere length, where older fathers produce offspring with longer telomeres [40,42,43,44,45]. These results are in favour of the hypothesis that telomeres are an epigenetic feature.

Although we are gaining insight into several aspects of the length of telomeres in the context of male reproduction, no study to date has related the effects of the phase of spermatogenesis and epididymal sperm maturation to telomere length with advancing paternal age, or established whether observed differences can be accounted for by the use of inbred and outbred rodent strains.

## 2. Materials and Methods

### 2.1. Animals

All studies were conducted on Brown Norway (BN) and Sprague Dawley (SD) transgenic rat strains bred in-house, with initial breeding pairs kindly provided by Dr. Hamra at UT Southwestern. The rats were transgenic for td-Tomato red (BN) and e-GFP (SD) expression in the germline. All animals had access to food and water ad libitum, and were kept in a 12-hour light, 12-hour dark, temperature- and humidity-controlled environment. BN and SD rats (n = 3–5) were sacrificed at young and aged time points. The average ages for the inbred BN rats were 5.6 months ± 0.2 and 19.2 ± 0.06 months for young and aged populations, respectively. For outbred SD rats, the average ages were 5.6 ± 0.18 and 18.7 ± 0.32 months for young and aged populations, respectively. Eighteen to twenty months of age in a rat is the age prior to germ cell loss and testicular atrophy [46]. Animal care and handling were done in accordance with the guidelines put forth by the Canadian Council on Animal Care (McGill Animal Resources Centre protocol 4687).

### 2.2. Germ Cell Separation

Young and aged rats were euthanized by CO_2_ asphyxiation. Testes were removed and weighed to assess the regression status associated with advanced aging, and rat testes less than 1.5 grams were considered regressed and not used in this study. When a testis was excluded, the attached epididymis was not used for sperm collection. Of 11 aged animals, four possessed only one testis that was not regressed. No animals had both testes regressed. Germ cells were obtained using the STA-PUT method for cell velocity sedimentation [47]. Briefly, testes were decapsulated prior to enzymatic digestion with 0.5 mg mL^−1^ collagenase (C9722-50MG; Sigma Aldrich, Oakville, Canada), followed by subsequent digestion with 0.5 mg mL^−1^ trypsin (Type I, T8003; Sigma-Aldrich, Oakville, Canada) and DNase I (Type I, DN-25; Sigma-Aldrich, Oakville, Canada). The dissociated germ cell suspension was then filtered through a 70 μM nylon mesh before being washed three times with 0.5% bovine serum albumin (A4612; Sigma Aldrich, Oakville, Canada) in RPMI 1640 (Life Technologies, Grand Island, USA) and pelleted at 233 g for 5 min. Cells were filtered once more with a 55 μM mesh to prevent clumping and 5.5 × 10^8^ mixed germ cells in 25 mL of 0.5% BSA in RPMI were loaded into the STA-PUT (Proscience, Toronto, Canada) and separated on a gradient of 2–4% BSA/RPMI. The gradient was established over 50 min, and the cells were separated through unit gravity sedimentation for 1 h 45 min. A fraction collector was then used to collect the germ cells in individual populations of pachytene spermatocytes (PS), and round (RS) and elongating spermatids. Fractions that met at least 80% purity by phase-contrast microscopy identification were spun down, flash frozen, and kept at −80 °C for future experiments. Spermatozoa from the caput and cauda epididymidis were isolated in PBS after 2 h of agitation. They were filtered through a 100 μM nylon mesh before being centrifuged and washed six times with 0.45% saline solution.

### 2.3. Telomere Measurement

DNA was extracted from 1.5 × 10^6^ PS, RS, and spermatozoa from the caput and cauda epididymidis using the QiaAMP DNA mini kit (51304; Thermo Fisher Scientific, Mississauga, Canada), with the substitution of a separate sperm lysis buffer including 40 mM dithiothreitol (DTT). Extracted sperm DNA may have different recoverability at the telomeres, as in these regions, it is packaged primarily with histones, while the remainder of the DNA is bound to protamine. Given our protocol for sperm DNA extraction, which disrupts the bound protamines, we did not anticipate that this would be an issue. DNA was diluted to a working concentration of 5 ng μL^−1^ for telomere measurement by qPCR [48] for telomeric repeats and 36B4 single copy gene amplification measured by ∆Ct. The mastermix for a final reaction volume of 20 μL per well was prepared using 10 μL per reaction SYBR Green MM solution (4367659; Thermo Fisher Scientific, Mississauga, Canada). For each 36B4 reaction, 1 μL of 2 μM forward and reverse primers for 36B4 and 5 μL PCR grade water were used. For each telomeric DNA reaction, 0.5 μL 2 μM forward and reverse primers for telomeric DNA with 4.5 μL of PCR grade water were used (Table S1). For all reactions, 20 ng/well DNA was used. The standard curve for telomeric repeats follows a 1:5 dilution, beginning with 4000 picograms (pg) of telomere oligomer (Table S2), corresponding to 7.6 × 10^9^ kb. The 36B4 standards begin with a concentration of 2 pg (Table S2), following a 1:10 dilution, corresponding to 3.6 × 10^9^ genome copies. Standards were brought to a total of 20 ng of DNA by spiking with pBR322 DNA. All samples presented herein fall along the presented standard curves. A four-step PCR amplification protocol was used. First, denaturation occurred at 95 °C for ten minutes (one cycle), followed by 40 cycles of denaturation at 95 °C for 15 s and annealing at 60 °C for 1 min. The melt curve conditions were 95 °C for 15 s, and annealing at 60 °C for 1 min, with a temperature increase of 0.5 °C per cycle to 95 °C for 15 s. The final step was an infinite hold at 4 °C. By taking the telomeric repeats, relative to the genome copies, the telomere length per genome was represented. DNA taken from H1301 cells (#01051619-DNA-5UG; Sigma-Aldrich, Oakville, Canada) with a known telomere length of 70 kb was then used for the normalization of all samples.

The calculations are as follows:

- Calculating telomeric repeats on a log scale:

(1) $$\log\left(Tel\right)=\frac{\Delta Ct-B}{m}$$

where the telomere standard curve produces the following slope: ΔCt=m (logTel)+B.

- Calculating genome copies (GC) represented by single copy gene 36B4:

(2) $$\log\left(GC\right)=\frac{\Delta Ct-B}{m}$$

where the 36B4 standard curve produces the following slope: ΔCt=m (logGC) + B.

- Calculating telomeric repeats per genome (telomere/single copy gene):

(3) $$\log\left(telomeric\;repeats\;per\;genome\right)=\frac{\log\left(Tel\right)}{\log\left(GC\right)}$$

(4) $$telomeric\;repeats\;per\;genome=\log{\left(telomeric\;repeats\;per\;genome\right)}^{10}$$

- Calculating telomere length relative to H1301 cell DNA, with a predicted telomere length of 70 kb:

(5) $$70\;kb=\frac{telomeric\;repeats\;pet\;genome\;H1301}{x}$$

(6) $$relative\;telomere\;length=\frac{telomeric\;repeats\;per\;genome\;}{x}$$

All experiments were done in triplicate, with intra-class correlation coefficients of 0.82 and 0.85 for young and aged BN sperm telomere lengths, respectively. To control for inter-plate variation, H1301 and standard curves were run on each plate. An inherent limitation of this protocol is the normalization of samples to H1301, as different methods of DNA extraction and handling can alter the apparent measure of telomere length. Though this was controlled for with samples processed in house, H1301 DNA was extracted and purified by Sigma.

### 2.4. Statistical Analysis

To calculate the telomere length, telomere kb and 36B4 genome copies were extrapolated from the standard curves and ΔCt values (Equations (1) and (2)). The telomere kb was divided by the genome copies represented by 36B4 (Equation (3)). These values were then normalized to the positive control H1301 DNA (Equation (4)), with a known telomere length of 70 kb, to give a measurement of relative telomere length (rTL). The median and interquartile range were calculated in Excel. Further statistics and data analysis were conducted using Graph-Pad Prism 6. Where appropriate, t-tests were used for statistical comparisons between groups; however, where variances were significantly different, a Mann Whitney U test was used as a replacement. Statistical significance of *p* ≤ 0.05 has been indicated with an asterisk (*).

## 3. Results and Discussion

### 3.1. Telomere Dynamics Show Rat Strain Specificity Between Brown Norway and Sprague Dawley Rats

**Figure A1:**
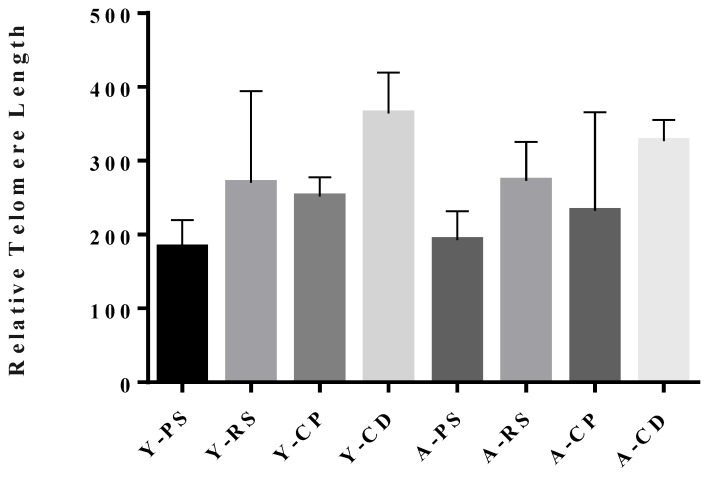
Relative telomere length (rTL) for the Sprague Dawley germline during aging. rTL for PS: Pachytene Spermatocyte, RS: Round Spermatid, CP: Caput Sperm, and CD: Cauda Sperm. Young samples presented as Y- (cell type) and aging samples presented as A- (cell type).

Telomere length for the outbred SD rats is in the range of 200–350 across spermatogenesis, while that for the inbred BN rats is shorter and has a decreased range of 115–160. Comparative studies of germ cell telomere length across varying species and strains have not been conducted. Although one would anticipate less variance in the lengths of telomeres from an inbred than outbred strain due to decreased genetic heterogeneity, it has also been proposed that inbred strains may have shorter somatic telomeres due to the increased oxidative stress and reduced evolutionary fitness [49]. The fact that this trend is maintained in the germline reveals potential long-term effects in an inbred rat strain as sperm telomere length is correlated with offspring telomere length [40,42,43,44,45]. Both strains show no difference in PS or RS rTL, a trending decrease in the caput sperm, and the subsequent recovery of telomere length in sperm from the cauda epididymidis. The most striking difference between strains is that both the interquartile range (IQR) and standard errors calculated for BN sperm telomere lengths are much smaller than those for the SD sperm (Table 1). The interquartile range represents the spread of data, by showing where 50% of the data points lie in a given sample set. The smaller IQR values for BN rats are likely due to the inbred nature of BN rats and the level of their genetic similarity. The homogeneity in rTL further validates them as a model for epigenetic studies in rodents. With both the inherent variability seen in the SD telomere length measurements and the exclusive use of BN rats for epigenetic studies, data for BN rats will be presented throughout the remainder of the text. The SD data is presented in Figure A1.

### 3.2. Telomere Lengths During Spermatogenesis in Brown Norway Rats

**Figure 1:**
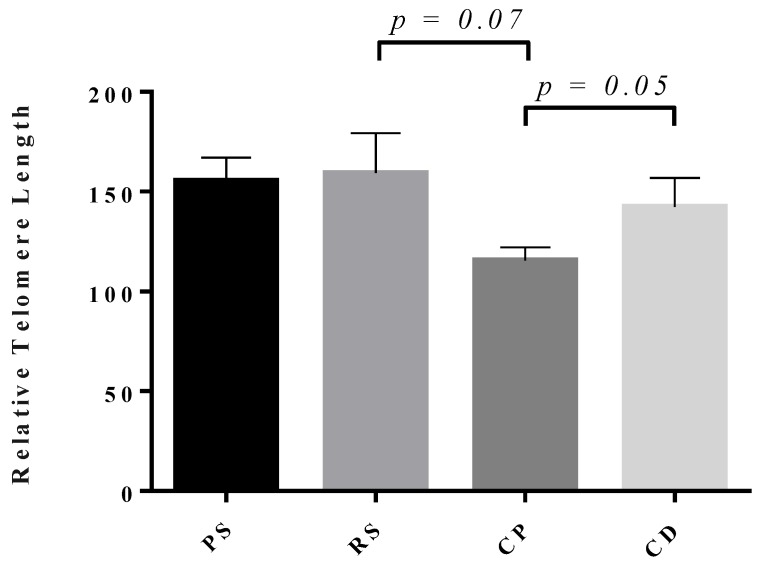
Telomere length in the young Brown Norway male germline. Relative telomere length (rTL) shown on the y-axis measured by quantitative polymerase chain reaction (qPCR) relative to H1301 cell DNA of a known telomere length, for pachytene spermatocytes (PS), round spermatids (RS), caput sperm (CP), and cauda sperm (CD). Each bar represents the mean ± SEM, n = 5. Sprague Dawley data shown in Figure A1.

Examining germ cell telomere dynamics has been done extensively in the context of telomerase activity, with a well-defined pattern of high telomerase activity in early germ cells that tapers off as spermatogenesis progresses. However, the existing literature that examines telomere length is less complete, mainly examining fully mature sperm and operating under the assumption that germ cell telomere length is strongly correlated with telomerase activity. When measuring rTL in BN rats, we find that there is no significant difference in the telomere length from PS to RS, with lengths measured at 155.4 (±11.6) and 159.2 (±20.1), respectively (Figure 1). This observation suggests that the length of telomeres remains relatively constant throughout the meiotic stages of spermatogenesis, independent of the apparent increase in telomerase activity [4]. An important component of understanding telomere dynamics throughout spermatogenesis that is missing is the measurement of telomere length in the spermatogonial stem cells; however, a methodology for the isolation of rat spermatogonial stem cells has yet to be developed.

Interestingly, when entering the epididymis, the length of telomeres shows a decrease of approximately 25% from what is observed for earlier stages of spermatogenesis. The length of telomeres from the spermatozoa obtained from the caput epididymidis of any species has not been measured previously, so it is difficult to determine if this novel observation can be generalized beyond the rat. This finding suggests altered telomere organization during chromatin condensation and crosslinking through epididymal maturation. However, what is apparent is that by the time sperm reach the cauda epididymidis, the sperm telomere length reaches a length of 142 (±14.6), comparable to the germ cell telomere length prior to entering the epididymis (Figure 1). The epididymis is a tissue that has received relatively little attention; understanding how the environment of the caput, corpus, and cauda epididymidis alters sperm chromatin is a major challenge that needs to be addressed by the scientific community. It is possible that telomere organization is impacted by micro and non-coding RNAs that are passed to the sperm through epididymosomes [50,51]. As more interactions are being elucidated for non-coding RNAs and telomeric regions, the functional role of these interactions will become clearer [52]. Telomeric repeat containing RNA (TERRA) is a non-coding RNA transcribed from telomeric regions that is able to bind telomeric DNA. The proposed function of TERRA binding is to control telomere structure and elongation; this has been shown in various species [53,54,55,56]. TERRA has also been shown to modify polycomb repressive complex binding, and modify histone marks across the genome and in telomeres [57]. Though there is limited literature on TERRA in male germ cells, Reig-Viader et al. have shown that it is present in spermatocytes and spermatids [58]. They have also shown that telomeres and TERRA levels were disrupted in germ cells from men with idiopathic infertility [59]. Taken together, these observations indicate the need for further studies to resolve the effects of non-coding RNAs during epididymal maturation.

### 3.3. Age-Dependent Decrease in Sperm Telomere Length

**Figure 2:**
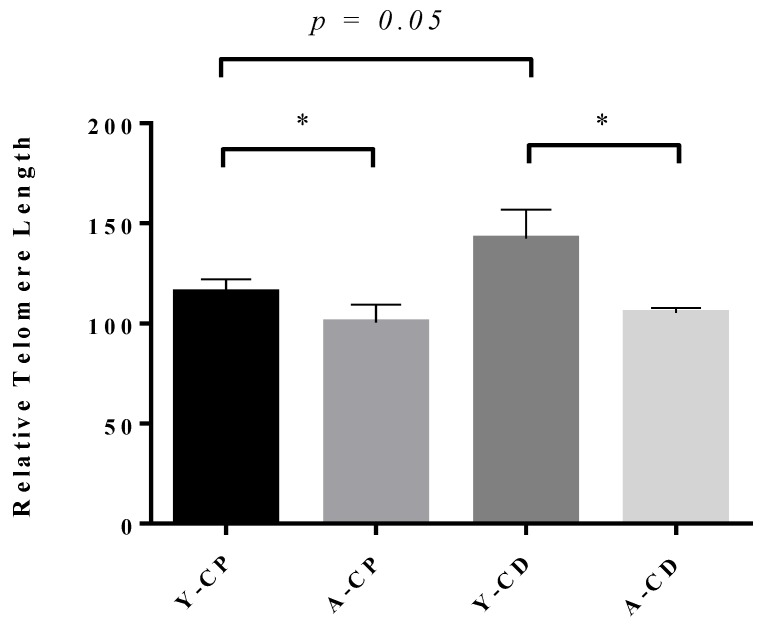
Telomere length for Brown Norway sperm during aging. Relative telomere length (rTL) shown on the y-axis measured by quantitative polymerase chain reaction (qPCR) relative to H1301 cell DNA of a known telomere length, for young caput sperm (Y-CP; n = 5), aged caput sperm (A-CP; n = 4), young cauda sperm (Y-CD; n = 5), and aged cauda sperm (A-CD; n = 4). Each bar represents the mean ± SEM. *p* ≤ 0.05 is indicated by an asterisk. Sprague Dawley data shown in Figure A1.

There is a significant age-dependent decrease in rTL from 115.6 (±6.5) to 93.3 (±6.3) in caput sperm (*p* = 0.04), which remained consistent for cauda sperm, with a decrease observed from 142.4 (±14.6) to 105.3 (±2.5) in cauda sperm (*p* = 0.02; Figure 2). This decrease is consistent with mouse models of paternal aging presented in the literature [38]. Interestingly, the trend for increased telomere length during epididymal transit is seemingly reduced with aging. A modest increase in rTL is observed from 93.3 (±6.3) to 105.3 (±2.5) in the caput sperm. If, during epididymal transit, non-coding RNAs contribute to affecting telomere length, it is possible that the epididymosome payload changes with advancing age, though no study to date exists on epididymosomes and aging.

There are currently no hypotheses to address the decrease in telomere length observed in rodent models of paternal aging. However, it seems probable that hypotheses proposed to explain germ cell telomere lengthening in humans may not apply to the much shorter lifespan of a rodent.

## 4. Conclusions

Understanding telomere length in the varying contexts that influence male reproductive function and spermatogenesis is critical to understanding their epigenetic implications. As telomeres are associated with the nuclear envelope, it remains plausible that they are sites initially recognized by the egg after fertilization to aid in chromatin anchoring; telomere length may also influence offspring health in this way [20]. Altered telomere length, either increased or decreased, may lead to a disruption in chromatin reorganization events following fertilization [28]. Studies by our group have shown several effects of aging on male reproductive outcomes, including increased time to pregnancy, higher resorption rates, and an increased instance of infertility [46]. It is difficult to conclude if the negative outcomes are associated with one specific pathology of aging, such as telomere length, as these cells are also exposed to increased oxidative stress and decreased DNA damage repair, and thus show increased DNA damage. The presence of increased DNA damage with aging has not been examined within telomeric regions; however, it may provide additional insight into sperm telomere dynamics during aging. Here, we have shown that sperm telomere length decreases with age in inbred Brown Norway rats. This poses an interesting question, and by examining telomere dynamics in embryos fertilized with young and aged sperm, we may begin to understand this relationship more clearly. Additionally, using RNA sequencing and chromatin conformation capture methods will elucidate how telomere dynamics are altered across spermatogenesis with aging.

## Figures and Tables

**Table 1:** Species variation for telomere length measurement in sperm.

Rat Strain	Cell Type	N	Median rTL	IQR	SEM
SD - Young	PS	5	205.89	126.14	37.21
	RS	4	180.53	141.46	124.19
	CP	6	230.47	79.17	25.54
	CD	10	396.81	253.57	54.94
SD - Aged	PS	6	204.92	132.51	38.99
	RS	5	301.42	122.45	52.70
	CP	3	116.93	205.51	133.00
	CD	12	302.82	132.82	28.49
BN - Young	PS	5	143.70	13.35	11.63
	RS	5	165.79	77.18	20.09
	CP	5	116.61	17.56	6.49
	CD	5	129.67	17.42	14.61
BN - Aged	CP	4	97.47	13.40	6.35
	CD	4	106.54	4.64	2.52

Species differences in relative telomere length (rTL) variability shown between Sprague Dawley (SD) and Brown Norway (BN) rats for pachytene spermatocytes (PS), round spermatids (RS), and sperm taken from the caput (CP) and cauda (CD) epididymis for both young and aged samples. N: Sample Size. IQR: Interquartile Range. SEM: Standard Error of the Mean.

## Supplementary Materials

The following are available online at https://www.mdpi.com/2073-4425/10/7/525/s1: Table S1: Telomere Length Quantitative Polymerase Chain Reaction (qPCR) Primers, Table S2: Telomere Length Quantitative Polymerase Chain Reaction (qPCR) Oligomer Standard Sequences.
