# Supplementary material for: Telomere Dynamics Throughout Spermatogenesis
Source: Genes (Basel). 2019 Jul 12;10(7):525. doi: 10.3390/genes10070525 (PMC6678359; doi:10.3390/genes10070525)
Supplement: Supplementary file 1 [file genes-10-00525-s001.pdf]

Table S1. Relative Telomere Length qPCR Primers

Repeat Region	Forward Primer	Reverse Primer
Telo	CGGTTTGTTTGGGTTTGGGTTTG GGTTTGGGTTTGGGTT	GGCTTGCCTTACCCTTACCCTTACCCTTAC CCTTACCCT
36B4	CGACCTGGAAGTCCAACTAC	ATCTGCTGCATCTGCTTG

Table S2. Relative Telomere Length qPCR Oligomer Standard Sequences

Standard	Oligomer Sequence
Telomere	$(TTAGGG)^{14}$
36B4	CGACCTGGAAGTCCAACTACTTCCTTAAGATCAT CCAACTTTTGGATGACTACCCAAAATGCTTCATT GTGGGAGCAGACAATGTGGGCTCCAAGCAGATG CAGCAGA
